# Perioperative haemostasis with full‐length, PEGylated, recombinant factor VIII with extended half‐life (rurioctocog alfa pegol) in patients with haemophilia A: Final results of a multicentre, single‐arm phase III trial

**DOI:** 10.1111/hae.13807

**Published:** 2019-07-28

**Authors:** Ralph Gruppo, Maria‐Fernanda López‐Fernández, Tung T. Wynn, Werner Engl, Marlies Sharkhawy, Srilatha Tangada

**Affiliations:** ^1^ Cincinnati Children’s Hospital Medical Center Cincinnati Ohio; ^2^ Complejo Hospitalario Universitario A Coruña Coruña Spain; ^3^ College of Medicine University of Florida Gainesville Florida; ^4^ Baxalta Innovations GmbH, a Takeda company Vienna Austria; ^5^ Baxalta US Inc., a Takeda company Cambridge Massachusetts USA

**Keywords:** BAX 855, extended half‐life recombinant factor VIII, haemophilia A, perioperative haemostasis, rurioctocog alfa pegol, TAK-660, surgery

## Abstract

**Introduction:**

Rurioctocog alfa pegol (BAX 855, TAK‐660) is a PEGylated, full‐length, recombinant factor VIII (rFVIII) with extended half‐life developed from unmodified rFVIII (antihaemophilic factor [recombinant]).

**Aim:**

To determine the perioperative haemostatic efficacy and safety of rurioctocog alfa pegol in male previously treated patients (PTPs) with severe haemophilia A.

**Methods:**

This multicentre, single‐arm, phase III study included PTPs who were to undergo major or minor elective or minor emergency surgical, dental or other invasive procedures. Rurioctocog alfa pegol dose and frequency were individualized based on patients’ pharmacokinetic profiles for major surgeries and by rurioctocog alfa pegol incremental recovery for minor surgeries. Haemostatic efficacy was assessed using the Global Haemostatic Efficacy Assessment score.

**Results:**

Twenty‐one patients aged 16‐61 years underwent 21 major and five minor surgeries. For all 24 evaluable surgeries, overall haemostatic efficacy was rated as excellent and blood loss comparable to that expected in non‐haemophilic patients. No blood transfusions were required intraoperatively but were administered postoperatively for four surgeries in three patients. Five injury‐related postoperative bleeding episodes occurred in five patients, of which two required additional rurioctocog alfa pegol treatment. Two non‐serious adverse events of mild severity (increased ALT level and headache) were considered possibly related to rurioctocog alfa pegol. There were no deaths or treatment‐related serious adverse events. No patients developed inhibitory antibodies to FVIII or persistent IgG‐ or IgM‐binding antibodies to FVIII, PEG‐FVIII or PEG.

**Conclusion:**

Rurioctocog alfa pegol was well tolerated and effective for perioperative use in patients with haemophilia A and showed no signs of immunogenicity.

## 1 INTRODUCTION

Haemophilia A is a deficiency in clotting factor VIII (FVIII) inherited in an X‐linked manner, almost invariably presenting in males. It increases the risk of acute bleeding within joints leading to arthropathy^1^ and also increases healing time after surgery or trauma.^2^ As a result of improvements in treatment, life expectancy among patients with haemophilia in developed countries is approaching that of the general population.^3^ This has resulted in an increase in age‐related conditions including requirement for surgery in this population.^4^, ^5^, ^6^, ^7^ In addition, joint surgery is frequently required for pain or disability arising from haemophilic arthropathy.^4^ Intensified FVIII replacement therapy is required in patients with severe haemophilia A (FVIII <1%) during and after surgery until healing is complete, for up to 7 days or more following major surgery.^8^ Because of their relatively short half‐life (approximately 12 hours), standard FVIII formulations require administration twice or three times daily to maintain haemostatic FVIII levels in the postoperative period. An extended half‐life FVIII may offer less frequent dosing and the possibility of earlier hospital discharge and attendant cost savings.^9^, ^10^, ^11^

Rurioctocog alfa pegol (BAX 855, TAK‐660; ADYNOVATE^®^, ADYNOVI™, Baxalta US Inc., a Takeda company, Lexington, MA, USA) is a PEGylated, full‐length, recombinant FVIII (rFVIII) with extended half‐life, developed from unmodified rFVIII (antihaemophilic factor [recombinant]; ADVATE^®^, Baxalta US Inc., a Takeda company, Lexington, MA,USA).^12^, ^13^ Mean half‐life of rurioctocog alfa pegol is 1.3‐ to 1.5‐fold longer in children aged <12 years and 1.4‐ to 1.5‐fold longer in adolescents and adults aged ≥12 years compared with its non‐PEGylated parent rFVIII.^12^, ^14^ Rurioctocog alfa pegol has been shown to be effective and well tolerated in the prevention and control of bleeding in previously treated paediatric and adult patients with severe haemophilia A.^12^, ^14^ The aim of our study was to determine the perioperative haemostatic efficacy and safety of rurioctocog alfa pegol in male previously treated patients (PTPs) with severe haemophilia A. Interim results from a prospectively planned analysis have been published^15^; here, we report the final results of this study.

## 2 MATERIALS AND METHODS

The study was performed in accordance with Good Clinical Practice and ethical principles consistent with the Declaration of Helsinki and was registered at http://clinicaltrials.gov (NCT01913405) and at http://clinicaltrialsregister.eu (2013‐001359‐11). The protocol was approved by the independent review boards at each participating centre. Written informed consent was provided by each patient before recruitment.

### 2.1 Objectives

The primary objective of the study was to determine the perioperative haemostatic efficacy of rurioctocog alfa pegol in male PTPs (≥150 prior exposure days) with severe haemophilia A undergoing major or minor elective or minor emergency surgical, dental or other invasive procedures, as determined by the Global Haemostatic Efficacy Assessment (GHEA) score. Secondary objectives included intra‐ and postoperative blood loss; volume of blood, red blood cells, platelets, and other blood products transfused; occurrence of bleeding episodes and additional need for surgical intervention; daily and total weight‐adjusted consumption of rurioctocog alfa pegol; and safety, as previously detailed.^15^

### 2.2 Study design

This was a phase III, prospective, open‐label, single‐group, multicentre study to evaluate the efficacy and safety of rurioctocog alfa pegol in PTPs undergoing major or minor elective or minor emergency surgical, dental or other invasive procedures. Surgical procedures were prospectively defined as major or minor by the investigator/surgeon based on protocol guidance as previously detailed^15^ in accordance with international guidelines.^8^, ^16^

Adjunct antifibrinolytic agents, for example tranexamic acid, or topical haemostatic agents were permitted. Mechanical thromboprophylaxis was allowed, and pharmacological thromboprophylaxis was permitted for certain surgical interventions at the investigator's discretion.

### 2.3 Patients

The study included male patients with severe haemophilia A. Eligible patients could transition from another rurioctocog alfa pegol study or were newly recruited. If newly recruited, patients were to be ≥12‐75 years of age, receiving prophylaxis or on‐demand treatment with FVIII at study entry, had a documented exposure to FVIII of ≥150 days and had no detectable FVIII inhibitory antibodies (≥0.4 Bethesda units using the Nijmegen‐modified Bethesda assay). Major exclusion criteria were need for major emergency surgery; detectable or a history of FVIII inhibitory antibodies; platelet count <100 × 10^9^/L; ongoing or recent thrombotic disease; diagnosis of an inherited or acquired haemostatic defect other than haemophilia A; recent use of another PEGylated product; and incremental recovery (IR) <1.5 IU/dL:IU/kg determined during participation in another rurioctocog alfa pegol study or after screening in this surgery study.

### 2.4 Pharmacokinetic assessment

Presurgical pharmacokinetics of rurioctocog alfa pegol were determined after a 60 IU/kg dose and included IR; area under the plasma concentration time curve from time 0 to ∞ and from time 0 to 96 hours; mean residence time; clearance; terminal half‐life (*t*_1/2_); and volume of distribution at steady state. IR was also assessed following the initial preoperative bolus infusion, and throughout the study. The pharmacokinetic parameters were determined using non‐compartmental methods, except for *t*_1/2_. Blood samples were taken for pharmacokinetic evaluation of FVIII levels and activated partial thromboplastin time within 30 minutes preinfusion and 15 ± 5 minutes postinfusion. Additional blood samples were taken for FVIII activity levels at 3 hours ± 30 minutes, 9 hours ± 30 minutes, 32 hours ± 2 hours, 56 hours ± 4 hours and 96 hours ± 4 hours.

### 2.5 Treatment

Details of perioperative treatment with rurioctocog alfa pegol have been previously published.^15^ Briefly, the dose and frequency of rurioctocog alfa pegol were individualized based on patients’ pharmacokinetic parameters for major surgeries and the most recent IR value for minor surgeries. A loading dose was administered within 60 minutes before surgery to achieve FVIII target levels of 80%‐100% of normal for major surgery, and FVIII target levels of 30%‐60% of normal for minor procedures.^17^ For major surgery, FVIII trough levels were required to be maintained ≥80% for the first 72 hours and at least 50% on postoperative days 4‐7. From day 8 until discharge, the FVIII levels were to remain above 30% (at the discretion of the investigator, depending on the postoperative course). For minor surgery, FVIII trough levels were targeted postoperatively at 30%‐60% for the first 24 hours (or longer if deemed necessary by the investigator). For all surgeries, FVIII levels were not to exceed supraphysiological peak FVIII levels of 180%.

### 2.6 Assessment of haemostatic efficacy

The primary outcome measure used (ie the GHEA score), comprised three assessments of haemostatic efficacy: intraoperative performed by the operating surgeon on day 0, postoperative by the operating surgeon on postoperative day 1 and perioperative performed by the investigator at discharge or on postoperative day 14. Each assessment was scored on a 4‐point scale (0 = none, 1 = fair, 2 = good, 3 = excellent). Detailed criteria for the GHEA score have been previously published.^15^

Actual intraoperative and postoperative blood loss was compared with that estimated by the surgeon/investigator for the same surgical intervention in a haemostatically normal individual of the same sex, age and stature as the study patient. Estimates took into account all relevant variables, for example the use of a tourniquet, the placement of a postoperative drain and the use of suction.

### 2.7 Safety assessment

Safety outcomes assessed included thrombotic events, severe allergic reactions, other treatment‐related adverse events (AEs), and clinically significant changes in vital signs and laboratory parameters. Samples were investigated for inhibitory antibodies to FVIII, and development of binding antibodies to FVIII, rurioctocog alfa pegol, PEG and Chinese hamster ovary (CHO) proteins.

### 2.8 Statistics

The target sample size of approximately 50 major and minor surgeries in approximately 40 patients was based on regulatory guidance to evaluate a minimum of 10 major surgical procedures in at least five patients^18^ and was not based on statistical considerations. The results were summarized by descriptive statistics. Median values are reported with their range throughout.

## 3 RESULTS

### 3.1 Demographics and patient disposition

**Figure 1:**
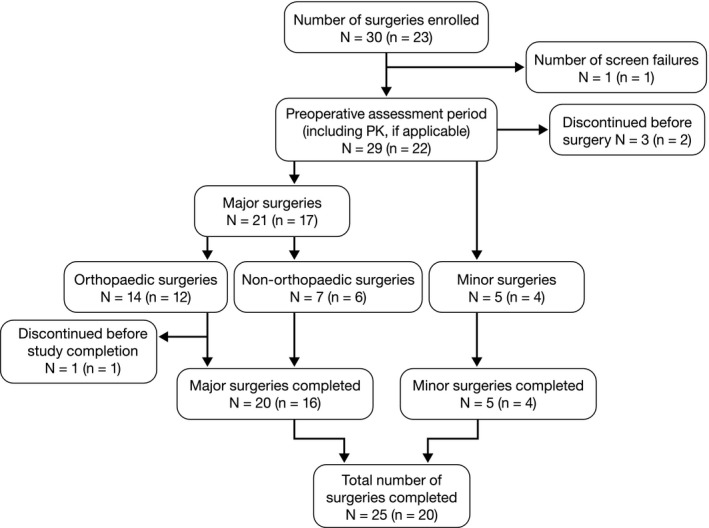
Patient disposition. Note: The numbers outside the parentheses are counted on surgical enrolments and those inside the parentheses are based on unique patients. One subject underwent both orthopaedic and non‐orthopaedic major surgery, which is therefore counted twice in the flowchart. PK, pharmacokinetics

The study was conducted between 20 December 2013 and 23 September 2016. Figure 1 shows the disposition of the patients and surgeries performed during the study. Twenty‐two patients were treated with rurioctocog alfa pegol and comprised the safety set. Patients’ demographic and clinical characteristics are summarized in Table 1; all were male, most were white (91%) and adult (96%), and had a history of haemophilic arthropathy (91%). Patients were recruited at 12 study sites in the United States (n = 4), Spain (n = 3), Bulgaria, Lithuania, Russia, Switzerland and the United Kingdom (each n = 1). One patient withdrew before surgery after receiving rurioctocog alfa pegol infusions for pharmacokinetic assessment. Twenty‐six surgeries (14 major orthopaedic, seven major non‐orthopaedic and five minor) were performed in 21 unique patients. Seven of the 14 major orthopaedic surgeries were arthroplasties. Among these, one patient underwent surgery but discontinued before study completion. Of the 21 unique patients, six patients (who underwent four minor and four major surgeries) were transitioned from another rurioctocog alfa pegol study^12^ and 15 patients (who underwent one minor and 17 major surgeries) were newly recruited. Seven patients received pharmacologic thromboprophylaxis after nine surgeries (three bemiparin plus rivaroxaban, two bemiparin, one enoxaparin and one heparin). Four patients undergoing four oral surgeries (three major, one minor) received antifibrinolytic therapy during surgery; two with tranexamic acid and one each with aminocaproic acid, and etamsylate plus tranexamic acid.

**Table 1:** Baseline clinical and demographic characteristics and type of surgery

Characteristic	Patients (N = 22)^a^	Type of Surgery	Surgeries (N = 26)^b^
Age at enrolment, y		Major	
Median (range)	33 (16‐61)	Orthopaedic	
<18	1 (5)	Knee replacement	3 (12)
18‐75	21 (96)	Arthroscopic synovectomy	3 (12)
Sex		Alloplastic knee surgery^c^	3 (12)
Male	22 (100)	Hip replacement	1 (4)
Race		Hip replacement revision	1 (4)
White	20 (91)	Elbow cyst extirpation	1 (4)
Black	1 (5)	Needle removed from elbow	1 (4)
Asian	1 (5)	Achilles tendon reconstruction	1 (4)
FVIII gene mutation		Non‐orthopaedic	
Inversion intron 22	4 (18)	Multiple tooth extractions	5 (19)
Frameshift	1 (5)	CVAD placement	1 (12)
Deletion	1 (5)	Gastric band insertion	1 (12)
Nonsense	1 (5)	Minor	
Point	1 (5)	Dermatological	2 (8)
Not known	14 (64)	Synoviorthesis	1 (12)
Arthropathy at screening		Dental procedure	1 (12)
Yes	20 (91)	Radiosynovectomy	1 (12)
No	2 (9)		

Note

Data are given as n (%) unless otherwise stated.

Abbreviation: CVAD, central venous access device.

^a^Safety population (N is number of unique patients).

^b^Full analysis set (N is number of surgical enrolments).

^c^Alloplastic knee surgery refers to any procedure to repair the knee joint using exogenous material, that is less extensive than a full knee replacement.

### 3.2 Primary efficacy outcome

Haemostatic efficacy for all 24 surgeries (21 major, three minor) with available GHEA scores was rated as excellent (Table 2). Intraoperative efficacy of rurioctocog alfa pegol assessed at the time of discharge from the operating room was excellent (blood loss ≤100% of that predicted preoperatively by the investigator for the type of procedure performed in a non‐haemophilic population) for all evaluable surgeries. Postoperative efficacy of rurioctocog alfa pegol assessed on postoperative day 1 was excellent (as defined above) for all evaluable assessments except one minor surgery, which was rated as good (mild injury‐related bleeding episode in the gum approximately 1 day after dental surgery). Perioperative efficacy, as assessed at discharge or day 14 (whichever was first), was excellent (blood loss ≤100% of that expected for the type of procedure performed in a non‐haemophilic population and blood components for transfusions less than or similar to that expected in a non‐haemophilic population) for all surgeries.

**Table 2:** Intraoperative, postoperative, perioperative and global haemostatic efficacy assessment scores

Type of surgery	N	Score	Intraoperative	Postoperative	Perioperative	Global
Major, orthopaedic	14	Excellent	14 (100%)	14 (100%)	14 (100%)	14 (100%)
Major, non‐orthopaedic	7	Excellent	7 (100%)	7 (100%)	7 (100%)	7 (100%)
Minor	5	Excellent	4 (80%)	3 (60%)	5 (100%)	3 (60%)
		Good	0	1 (20%)	0	0
		Not done	1 (20%)	1 (20%)	0	2 (40%)
All surgeries	26	Excellent	25 (96%)	24 (92%)	26 (100%)	24 (92%)
		Good	0	1 (4%)	0	0
		Not done	1 (4%)	1 (4%)	0	2 (8%)

Note

Full analysis set. N is number of surgical enrolments.

### 3.3 Secondary efficacy outcomes

Data for median intra‐ and postoperative blood loss are summarized in Table 3. Actual intraoperative blood loss for major orthopaedic surgeries was substantially less than the average volume predicted by the investigators (median, 125 mL less). Actual intraoperative blood loss was similar to the predicted average volumes for non‐orthopaedic major (median, 1.5 mL less) and minor (no difference) surgeries. Actual postoperative blood loss was higher than the average volume predicted for orthopaedic major surgeries (median, 50 mL more) but lower than the maximum volume predicted (median, 100 mL less). Actual postoperative blood loss was similar to the average volume predicted for non‐orthopaedic major (median, 4.0 mL less) and minor (no difference) surgeries.

**Table 3:** Intraoperative and postoperative blood loss

Period and parameter	Major surgeries (N = 21)		Minor surgeries (N = 5)	All surgeries (N = 26)
	Orthopaedic (N = 14)	Non‐orthopaedic (N = 7)		
Intraoperative				
Actual blood loss, mL^a^	10.0 (0‐250)	4.5 (1‐50)	5.0 (0‐50)	10.0 (0‐250)
Predicted average blood loss, mL^b^	150.0 (0‐500)	10.0 (2‐150)	5.0 (0‐200)	20.0 (0‐500)
Difference from predicted average blood loss, mL	125.0 (0‐308)	1.5 (0‐100)	0.0 (−45 to 195)	6.0 (−45 to 308)
Predicted maximum blood loss, mL^b^	300.0 (0‐2000)	20.0 (4‐250)	5.0 (0‐200)	100.0 (0‐2000)
Difference from predicted maximum blood loss, mL	275.0 (0‐1750)	25.0 (0‐200)	0.0 (−45 to 195)	100.0 (−45 to 1750)
Postoperative^c^				
Actual blood loss, mL^a^	750.0 (0‐1200)	1.0 (0‐65)	0.0 (0‐4)	10.0 (0‐1200)
Predicted average blood loss, mL^b^	213.5 (0‐700)	1.0 (0‐50)	0.0 (0‐200)	27.5 (0‐700)
Difference from predicted average blood loss, mL	−50.0 (−500 to 295)	4.0 (−15 to 25)	0.0 (0‐196)	−7.5 (−500 to 295)
Predicted maximum blood loss, mL^b^	450.0 (0‐1200)	2.0 (0‐150)	0.0 (0‐200)	75.0 (0‐1200)
Difference from predicted maximum blood loss, mL	100.0 (−15 to 595)	34.0 (0‐85)	0.0 (0‐196)	67.5 (−15 to 595)

Note

Full analysis set. N is number of surgical enrolments. Data presented as median (range).

a Actual blood loss determined by drainage volume, if applicable, and the estimated blood loss into swabs and towels during the procedure. Surgeries for which estimates of actual blood loss were available: intraoperative period 14 (major orthopaedic), six (major non‐orthopaedic), five (minor), 25 (all); postoperative period nine (major orthopaedic), four (major non‐orthopaedic), three (minor), 16 (all).

b Preoperative prediction by surgeon/investigator (data available for all surgeries).

c From completion of procedure until 24 h postsurgery.

No blood transfusions were required intraoperatively. Five transfusions of packed red blood cells (PRBCs) were administered postoperatively for four surgeries in three patients (three transfusions for three major orthopaedic surgeries [all joint surgeries] in two patients and two transfusions for a single major non‐orthopaedic surgery [gastric band insertion]). All transfusions were indicated for low haemoglobin. The median volume of PRBCs transfused per surgery with transfusions was 430 (range, 293‐600) mL.

Five bleeding episodes were reported in five unique patients, which were classified as mild (n = 3), moderate (n = 1) and severe (n = 1). Two mild and one moderate bleeding episodes that did not require additional treatment with FVIII products included mild mucosal bleeding 1 day after minor dental surgery, mild mucosal bleeding ~19 hours after major non‐orthopaedic surgery with central venous access device placement and moderate gastrointestinal bleed 1 day after major abdominal surgery for gastric band insertion. The mild bleeding in the left knee approximately 1 month after major orthopaedic surgery with a knee replacement and the severe bleeding episode (bleed in the musculus iliopsoas ~1 week after major orthopaedic surgery for arthroscopic tarsus synovectomy) both required treatment with rurioctocog alfa pegol to successfully control bleeding. All bleeding episodes were categorized as injury‐related; none were spontaneous or of unknown cause. None of the 26 surgical enrolments had an additional need for a surgical intervention.

### 3.4 Rurioctocog alfa pegol dosage and consumption

**Figure 2:**
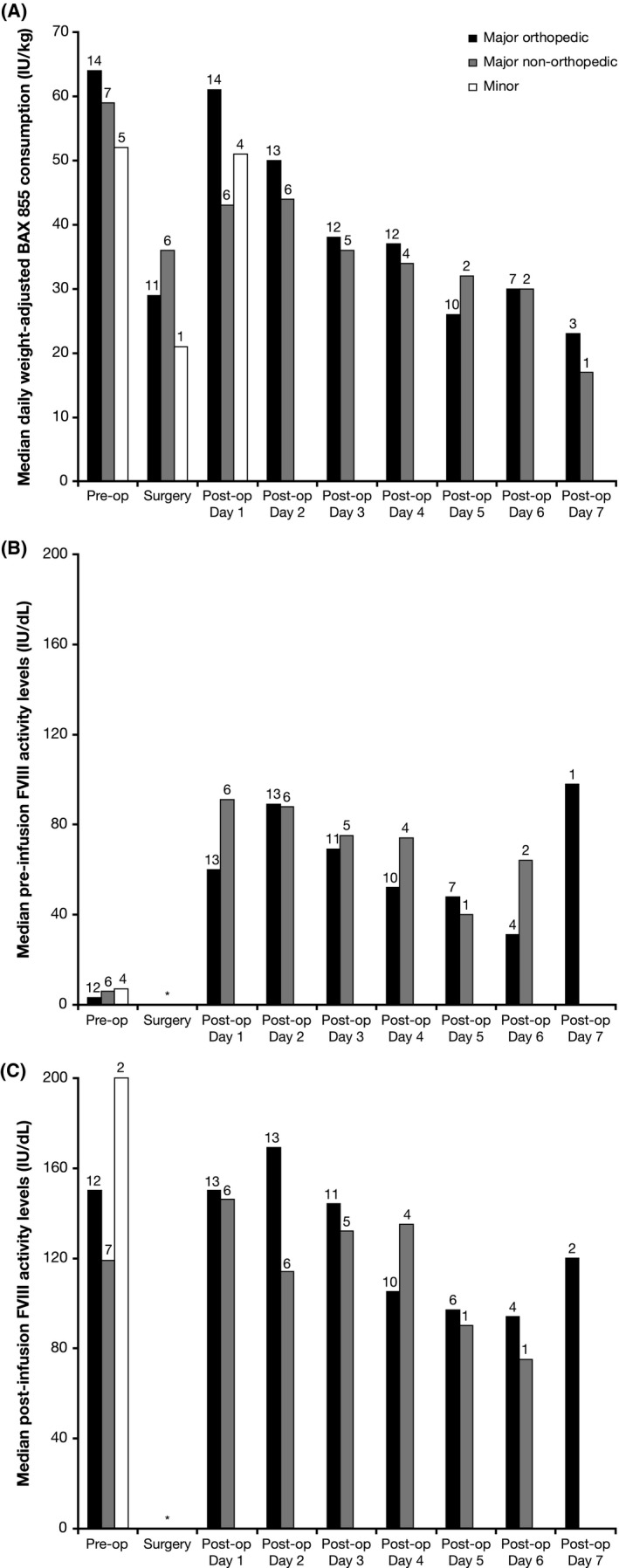
A, Median daily weight‐adjusted rurioctocog alfa pegol consumption (IU/kg); B, Median trough (30 min prior to infusion) FVIII activity levels (IU/dL); and C, Median peak (15 min postinfusion) FVIII activity levels (IU/dL); all according to type of surgery. N values appear above each bar. *Trough and peak FVIII activity levels are not available

The median (range) preoperative loading dose of rurioctocog alfa pegol was 64 (51‐99) IU/kg for orthopaedic major surgeries, 59 (36‐77) IU/kg for non‐orthopaedic major surgeries and 52 (39‐70) IU/kg for minor surgeries. The median (range) total dose of rurioctocog alfa pegol per patient was 629 (464‐1457) IU/kg for major orthopaedic surgeries, 489 (296‐738) IU/kg for major non‐orthopaedic surgeries and 120 (104‐151) IU/kg for minor surgeries. Daily weight‐adjusted consumption of rurioctocog alfa pegol before discharge from hospital across all surgeries is displayed in Figure 2A. The median daily weight‐adjusted consumption of rurioctocog alfa pegol was generally similar for major orthopaedic and non‐orthopaedic surgeries pre‐, intra‐ and postoperatively. FVIII activity levels 30 minutes preinfusion (trough) and 15 minutes after infusion (peak) for all surgeries are shown in Figure 2B and 2C, and generally declined from postoperative day 1 through to day 7, in keeping with weight‐adjusted consumption.

### 3.5 Pharmacokinetics

Presurgical pharmacokinetics were determined for the 25 surgeries in 20 unique patients for whom data were available, following a median rurioctocog alfa pegol dose of 60 (range, 51‐67) IU/kg [Table 4]). Median IR values throughout the study (preoperatively, on postoperative days 1, 2, 3, 4, 5, 6, 7 and 14, and at discharge) ranged from 1.6 to 2.2 IU/dL:IU/kg.

**Table 4:** Summary of presurgical rurioctocog alfa pegol pharmacokinetic parameters

Parameter	Median (range)
AUC_0‐96 h_, IU·h/dL	2704 (1382‐4533)
AUC_0‐∞_, IU·h/dL	2725 (1383‐4654)
*t*_1/2_, h	14.2 (8.8‐22.3)
MRT, h	19.6 (10.3‐29.9)
CL, dL/kg·h	0.021 (0.013‐0.043)
*V*_SS_, dL/kg	0.428 (0.271‐0.682)
IR at 15 min postinfusion, (IU/dL)/(IU/kg)^a^	2.04 (1.59‐3.15)
IR at *C*_max_, (IU/dL)/(IU/kg)	2.05 (1.48‐3.15)

Note

Pharmacokinetic analysis set. Data were generated using the one‐stage clotting assay. The pharmacokinetic population for analysis included 25 surgeries in 20 unique patients.

Abbreviations: AUC_0‐96 h_, AUC from time zero to 96 h; AUC_0‐∞_, AUC from time 0 to ∞; CL, clearance; *C*_max_, maximum plasma concentration; IR, incremental recovery; MRT, mean residence time; *t*_1/2_, terminal half‐life; *V*_SS_, volume of distribution at steady state.

a Data for 23 surgeries were included when a 15‐min postinfusion blood draw was originally planned.

### 3.6 Safety

Eighteen treatment‐emergent AEs were reported for eight (36%) unique patients: all but two of these AEs were considered unrelated to rurioctocog alfa pegol by the investigators. Two non‐serious AEs of mild severity (one increased alanine aminotransferase [ALT] level, one headache) were considered possibly related to rurioctocog alfa pegol. There were no AEs considered to be thrombotic events or related AEs considered to be allergic reactions. There were no treatment‐related serious AEs (SAEs) and no deaths.

Four non‐treatment‐related SAEs, one moderate and three severe, occurred in two patients. The three severe non‐related SAEs (oesophageal ulcer and two events of diabetic gastroparesis) occurred in one patient who received rurioctocog alfa pegol for the preinterventional pharmacokinetic assessment but did not undergo surgery. The moderate non‐related SAE (left hip prosthetic joint infection) occurred in a patient who underwent revision of the prosthetic.

None of the 22 patients developed inhibitory antibodies to FVIII, persistent IgG‐ or IgM‐binding antibodies to FVIII, PEG‐FVIII or PEG, or binding antibodies to CHO proteins. No trends over time were observed for clinical chemistry and haematology parameters. There were no abnormal findings in vital signs that were considered to be related to treatment with rurioctocog alfa pegol.

## 4 DISCUSSION

This first study of the perioperative use of rurioctocog alfa pegol demonstrated efficacy in most surgeries with no treatment‐related SAEs or development of inhibitory or persistent binding antibodies. This paper describes the final analysis of 26 surgeries in 22 subjects; a planned interim analysis of 15 surgeries in 15 patients was published in 2016.^15^ For all 24 surgeries evaluable for the primary outcome measure, overall haemostatic efficacy based on the GHEA score of assessments across intraoperative, postoperative and perioperative periods was rated excellent. GHEA scores were always classified as excellent except for one minor surgery classified as good postoperatively (mild injury‐related bleeding episode in the gum approximately 1 day after dental surgery). These results are similar to those observed in a study of unmodified, non‐PEGylated rFVIII in 58 patients undergoing 65 surgical procedures (including 22 major surgeries); haemostatic efficacy was rated good or excellent in all surgeries intraoperatively and at discharge.^19^

The median preoperative loading dose of rurioctocog alfa pegol was similar across the types of surgery performed (median 52‐64 IU/kg) while the median (range) total dose of rurioctocog alfa pegol per patient was 629 (464‐1457) IU/kg for major orthopaedic surgeries, 489 (296‐738) IU/kg for major non‐orthopaedic surgeries and 120 (104‐151) IU/kg for minor surgeries. For comparison, the median total consumption in the previous study of unmodified rFVIII for major (mostly orthopaedic) surgeries was 910 IU/kg (range 228‐1825) (bolus infusions only).^19^ There was a high variability in trough FVIII levels; however, medians were in the desired range and patients’ haemostatic efficacy ratings were excellent.

With respect to safety, there were no deaths, treatment‐related SAEs or thrombotic events. Two mild AEs (increased ALT level and headache) were considered possibly related to rurioctocog alfa pegol. No patients developed inhibitory antibodies to FVIII or persistent IgG‐ or IgM‐binding antibodies to FVIII, PEG‐FVIII, PEG or CHO proteins. The safety profile also appeared comparable to the parent unmodified rFVIII for perioperative haemostasis, where no treatment‐related SAEs occurred, only eight of 149 non‐serious AEs were thought possibly or probably related to study treatment, and no FVIII inhibitors were detected.^19^

One patient experienced a moderate SAE of left hip prosthetic joint infection following revision of the prosthesis, which was not considered related to treatment. Significantly lower postoperative infection rates have been observed in patients with haemophilia when postoperative FVIII activity levels are maintained at higher levels (≥80% over the first 2 postoperative weeks) than currently recommended by guidelines (120% at surgery down to 50% at 2 weeks postsurgery).^20^ Trough FVIII activity levels in this patient were as low as 40% 5 days after surgery, raising the theoretical possibility of an association with the infection. Thus, physicians may wish to consider maintaining higher‐than‐recommended FVIII activity levels following such surgeries.

With regard to sample size, regulatory guidance recommends that a minimum of 10 major surgical procedures in at least five patients are evaluated,^18^ and this requirement was surpassed in the current study with the enrolment of only 21 unique patients. Thus, further enrolment was unnecessary, and the target sample size was not reached.

In conclusion, rurioctocog alfa pegol was considered well tolerated and effective for perioperative use in PTPs with severe haemophilia A. The efficacy and safety results of rurioctocog alfa pegol in this study in the operative setting confirm those found in studies of rurioctocog alfa pegol in prophylactic and on‐demand settings^12^, ^14^ and were consistent with those for unmodified, non‐PEGylated rFVIII^19^ (from which rurioctocog alfa pegol was derived) in the perioperative setting.

## DISCLOSURES

RG received honoraria for participation in advisory boards for Baxter (Shire)* and Bayer. MFLF and TTW received honoraria as investigators in this study, which was sponsored by Shire.* MFLF has also received fees as a speaker and advisory board participant from Bayer, Baxalta/Shire*, Pfizer, LFB, Novo Nordisk, Amgen and Sobi. WE and MS are employees of Baxalta Innovations GmbH, a Takeda company. ST is an employee of Baxalta US Inc., a Takeda company, and a stock owner in Takeda. *A Takeda company.

## AUTHOR CONTRIBUTIONS

MFLF and TTW performed the research and acquisition of data. RG participated in the research and analysis of data. WE, MS and ST designed the study and analysed the data. All authors critically reviewed the manuscript and approved the final version.

## DATA AVAILABILITY

The datasets, including redacted study protocol, redacted statistical analysis plan and individual participants data behind the results reported in this article, will be available 3 months after the submission of a request to researchers who provide a methodologically sound proposal after de‐identification, in compliance with applicable privacy laws, data protection and requirements for consent and anonymization. Data requests should follow the process outlined in the Data Sharing section on Shire's website: http://www.shiretrials.com/en/our-commitment-to-transparency/data-sharing-with-researchers and should be directed to http://clinicaltrialdata@shire.com.
